# A Role for the Protein Tyrosine Phosphatase CD45 in Macrophage Adhesion through the Regulation of Paxillin Degradation

**DOI:** 10.1371/journal.pone.0071531

**Published:** 2013-07-31

**Authors:** Joëlle St-Pierre, Hanne L. Ostergaard

**Affiliations:** Department of Medical Microbiology and Immunology, and the Li Ka Shing Institute of Virology, University of Alberta, Edmonton, Alberta, Canada; UC San Diego, United States of America

## Abstract

CD45 is a protein tyrosine phosphatase expressed on all cells of hematopoietic origin that is known to regulate Src family kinases. In macrophages, the absence of CD45 has been linked to defects in adhesion, however the molecular mechanisms involved remain poorly defined. In this study, we show that bone marrow derived macrophages from CD45-deficient mice exhibit abnormal cell morphology and defective motility. These defects are accompanied by substantially decreased levels of the cytoskeletal-associated protein paxillin, without affecting the levels of other proteins. Degradation of paxillin in CD45-deficient macrophages is calpain-mediated, as treatment with a calpain inhibitor restores paxillin levels in these cells and enhances cell spreading. Inhibition of the tyrosine kinases proline-rich tyrosine kinase (Pyk2) and focal adhesion kinase (FAK), kinases that are capable of mediating tyrosine phosphorylation of paxillin, also restored paxillin levels, indicating a role for these kinases in the CD45-dependent regulation of paxillin. These data demonstrate that CD45 functions to regulate Pyk2/FAK activity, likely through the activity of Src family kinases, which in turn regulates the levels of paxillin to modulate macrophage adhesion and migration.

## Introduction

CD45 is a transmembrane PTP abundantly expressed on cells of hematopoietic origin [1]. It is a key regulator of Src family kinases (SFK), as it can both dephosphorylate the inhibitory and activation tyrosine residues of SFK, resulting in their hyperactivation or decreased activation, respectively [2], [3], [4]. The absence of CD45 from cells thus has important consequences in SFK-dependent functions of immune cells, including T- and B-cell receptor signalling [3]. Although the role of CD45 has been well established in lymphocytes, there are a limited number of studies that have investigated its role in leukocytes. One study has shown that the absence of CD45 from macrophages leads to the disregulation of macrophage adhesion [5]; however, the molecular mechanisms involved remain undefined.

CD45-dependent regulation of adhesion has been observed in T-cells [6], [7], [8]. Moreover, CD44-initiated spreading of T-cells involves the regulation of SFK and the cytoskeletal-associated protein proline-rich tyrosine kinase (Pyk2) by CD45 [8], [9], [10]. Pyk2 is a member of the focal adhesion kinase (FAK) family and is preferentially expressed in hematopoietic and neuronal cells [11]. This family of kinases, which also includes FAK, is involved in integrin-mediated cell adhesion and motility [11], [12]. Pyk2 is highly expressed in macrophages and contributes to adhesion, migration and polarization in response to integrin engagement [13], [14]. Macrophages isolated from Pyk2 KO mice are unable to polarize and migrate during chemotaxis *in vitro* and infiltrate inflammatory sites *in vivo* [15]. Pyk2-deficient macrophages also display defects in the contractile capacity of lamellipodia and have impaired F-actin localization [15]. Pyk2 has also been shown to localize to macrophage podosomes, where it colocalizes with the α_M_β_2_ integrin, as well as the cytoskeletal-associated proteins paxillin, vinculin and talin, and is phosphorylated upon α_M_β_2_ engagement [14]. Pyk2 and FAK share approximately 45% amino acid identity and 65% similarity [16], [17], [18], [19], [20]. These proteins have similar domain structure that includes an N-terminal FERM (Protein 4.1, Ezrin, Radixin, Moesin) domain, a centrally located kinase domain, two proline-rich regions in the C-terminus, along with a focal adhesion-targeting (FAT) domain [11], [12], [21]. The FERM domain contains an autophosphorylation site (Y402 in Pyk2, Y397 in FAK) that, upon phosphorylation, serves as a docking site for SFK [11], [22], [23]. Recruited SFK can then phosphorylate additional tyrosine residues within Pyk2 and FAK, leading to enhanced catalytic activity and docking sites for SH2 domain-containing proteins [12], [21], [24]. The FAT domain is necessary for interaction with the cytoskeletal-associated protein paxillin [25], [26].

Paxillin is a 68 kDa cytoskeletal-associated protein that acts as a scaffold for the coordination of protein signalling at sites of adhesion [27], [28]. The structure of paxillin reflects this role, as it contains multiple protein interacting domains. The N-terminus of paxillin contains five leucine- and aspartate-rich motifs (termed LD1 through 5) that are necessary for localization of paxillin to the actin cytoskeleton and for association with multiple cytoskeletal-associated proteins such as FAK, Pyk2 and vinculin, and the actin-binding protein actopaxin [25], [26], [29], [30]. A proline-rich region is found between LD1 and LD2 and is necessary for interaction with SH3 domain-containing proteins such as Src [31]. The C-terminus of paxillin contains four lin-11, isl-1, mec-3 (LIM) domains, which are double zinc-finger motifs that mediate protein-protein interactions. Phosphorylation sites contained in paxillin allow for tightly coordinated signalling events downstream of cellular adhesion. Phosphorylation of paxillin at Y31 and Y118 by FAK has been shown to regulate cell motility and allows for interaction with SH2-containing proteins such as SFK [32], [33], [34], [35]. The contribution of paxillin to cell adhesion and motility has been established in a variety of cell types [36]; however, few studies have examined its role in immune cells [30], [37], [38], [39], [40].

It is probable that the pathways leading to CD45-mediated regulation of macrophage adhesion may involve proteins controlled by SFK. Indeed, the SFK Hck and Lyn, but not Fgr, have been shown to be hyperphosphorylated and hyperactive in CD45 KO macrophages [5]. As Pyk2 and paxillin are downstream targets of SFK, and tyrosine phosphorylation of both these proteins have been shown to be disregulated in CD45-deficient T-cells [9], [41], it is reasonable to predict that these proteins participate in the CD45-dependent signalling pathways that control macrophage adhesion.

In this study, we have examined in detail the mechanisms by which CD45 regulates macrophage adhesion and motility. Previous studies on CD45 have focused on M-CSF-generated bone marrow-derived macrophages (BMDM) [5], [42]. In this current study, we have examined GM-CSF-generated BMDM, which tend to produce more inflammatory type cytokines [43], [44] and constitute a more heterogeneous population with increased cell spreading compared the smaller and more uniform M-CSF-derived BMDM [45], [46]. We show that GM-CSF-derived BMDM also display adhesion defects in the absence of CD45 and that this results in defective macrophage migration. We provide evidence that CD45 negatively regulates calpain-mediated degradation of paxillin in a Pyk2/FAK-dependent manner. This newly described regulatory mechanism provides further insight to the complexity of interactions between tyrosine phosphatases and kinases, cytoskeletal proteins and macrophage adhesion.

## Materials and Methods

### Ethic*s*

All animal experimental procedures were approved by the Health Sciences Animal Care and Use Committee at the University of Alberta (Protocol Number 055) and conform to guidelines put forward by the Canadian Council on Animal Care.

### Mice

B6.129ptprc^tm1-holmes^ (formerly known as CD45Δexon9 and referred to CD45−/− herein) [47] mice were purchased from The Jackson Laboratory (Bar Harbor, ME) and bred and housed in viral-antigen-free mouse facilities (Heath Sciences Lab Animal Services, University of Alberta). In order to insure similar genetic backgrounds between experimental mice, B6.129ptprc^tm1-holmes^ mice were crossed with C57BL/6 for six generations followed by the generation of separate CD45+/+ and CD45−/− colonies from genotyped offspring.

### Reagents and Antibodies

The monoclonal antibody to phosphotyrosine was purified from the PY72.10.5 hybridoma and is described elsewhere (Ostergaard et al, 1998). The paxillin monoclonal antibodies (clone 349, clone 165) were purchased from BD Transduction Laboratories (Mississauga, ON). Anti-Erk was acquired from Invitrogen (Camarillo, CA). Anti-GAPDH was obtained from Meridian Life Science (Saco, ME). Anti-calpain was purchased from Santa Cruz Biotechnology (Santa Cruz, CA). Anti-mouse-Rhodamine, HRP-conjugated anti-mouse and rabbit anti-mouse IgG were obtained from Jackson Laboratories. Phalloidin conjugated to fluorescein was bought from Invitrogen (Burlington, ON). Protein A sepharose beads were acquired from Amersham Biosciences (Piscataway, NJ). ALLN was obtained from Sigma-Aldrich (Mississauga, ON). The FAK and Pyk2 inhibitor PF431396 was purchased from Symansis (Shanghai, China). FBS was acquired from PAA Laboratories (Etobicoke, ON). The protease inhibitor cocktail tablets were acquired from Roche (Indianapolis, IN).

### Bone Marrow-derived Macrophage Isolation and Culture

Bone marrow was obtained by flushing tibiae and femurs of 12 to 15 week-old mice with PBS. Bone marrow precursor cells were cultured in bone marrow media (RPMI, 10% FCS, 2 mM L-Glutamine, 100 U/ml penicillin, 100 U/ml streptomycin, 0.053 µM β-mercaptoethanol) supplemented with 20% of filtered culture medium from GM-CSF-producing CHO cells (gift from the Dr. K.P. Kane, University of Alberta, Edmonton, AB). Progenitor cells were plated at 2×10^7^ cells per 20 mm tissue-culture-treated dish or 4×10^6^ cells per 10 mm tissue-culture-treated dish in media. Fresh media was added at day 3 and cells were cultured for a total of 7 days. Non-adherent cells were removed from the culture and discarded and adherent macrophages were harvested by gentle scraping. At this stage, over 80% of cells stained for F4/80 and over 90% stained for CD11b. Cells were then used for experimentation or lysed in at 10^7^ cells/ml of lysis buffer (1% Nonidet P-40, 10 mM Tris, 5 mM EDTA, 150 mM NaCl, 1 mM orthovanadate and protease inhibitors) for 20 minutes on ice followed by centrifugation at 13 000×g for 3 minutes to pellet out the nuclei. Post-nuclear lysates were used for immunoprecipitation or loaded onto SDS-PAGE gels for subsequent Western blotting.

### Adhesion Assays and Cell Treatment

To assess cell spreading in culture, adhered macrophages from day 7 cultures were washed and photographs were taken from three independent cultures. Cells (>1500 for each culture) were scored according to morphology and the presence or absence of visible adhesive structures on the cell contour. If cells were seen to have any extensions or protrusions that caused the cell to no longer have a round shape, they were scored as positive for cell spreading. To assess the effects of inhibitors on cell spreading, day 7 BMDM were washed on the plates and BMDM media was replaced serum-poor media (RPMI, 0.5% FCS) with the indicated amounts of inhibitors (ALLN, PF431396) or carrier control. Cells were incubated for 4 hours at 37°C. Quantification of cell spreading was done by scoring cells using the ImageJ software version 1.43 u (http://rsb.info.nih.gov). Cells were then lysed directly on the plate with reducing sample buffer and loaded on 8.5% SDS-PAGE gel for subsequent Western Blotting.

### Immunoprecipitation and Western blotting

For immunoprecipitation, post-nuclear lysates of 1×10^7^ cells were incubated with anti-paxillin for 15 minutes, followed by incubation rabbit anti-mouse IgG for 15 minutes and Protein A Sepharose beads (30 µl of 50% slurry) for 1.5 hours. Incubations were done at 4°C on a rotator. Beads were pelleted and washed three times with lysis buffer before resuspension in reducing sample buffer and boiled for 3 minutes. Total cell lysates or immunoprecipitates were loaded onto 8.5% SDS-PAGE gels followed by transfer to polyvinylidene difluoride (PVDF) membranes. Western blots were performed using the indicated primary and appropriate HRP-coupled secondary antibodies and visualized by ECL (PerkinElmer Life Science Products, Boston, MA). When sequential Western blots were performed on the same membrane, the membrane was stripped in buffer containing β-mercaptoethanol, SDS and Tris-HCl (pH 6.7) at 56°C in between each blot. The order of the blots in the figures is representative of the order in which the membranes were probed with the indicated antibodies. Quantification of Western blot bands was performed using the ImageJ software, version 1.43 u (http://rsb.info.nih.gov/ij/).

### Confocal Microscopy and Live Cell Imaging

Cells were harvested, washed and adhered onto Poly-L-lysine-coated coverslips for 15 minutes at room temperature. The coverslips were then washed once with PBS containing 1% FCS. The cells were fixed with 4% formaldehyde for 10 minutes at room temperature, and coverslips were washed twice with 1% FCS in PBS. The cells were permeabilized with 0.2% Nonidet P-40 in PBS for 5 minutes at room temperature and the coverslips were subsequently washed three times with 1% FCS in PBS. Cells were incubated in blocking buffer (1%FCS, 1% normal goat serum serum in PBS) for 30 minutes at room temperature. Coverslips were then incubated with each primary and fluorochrome-conjugated secondary antibody diluted in 1% FCS in PBS for 45–60 minutes at room temperature in a dark chamber. The coverslips were washed three times with PBS for 5 minutes after each antibody incubation. Coverslips were mounted on glass slides using mounting medium (PBS, 20% glycerol, 0.1% Mowiol, 0.1% propyl gallate). Samples were analyzed on a Zeiss LSM 710 confocal microscope with the 63X/1.40 oil objective (Imaging Centre, Cross Cancer Institute, Edmonton, AB).

For live cell imaging, day 7 BMDM were harvested, washed and incubated in wells for 2 hours prior to imaging. Cells were tracked for 30 to 45 minutes on an Olympus IX-81 Motorised microscope equipped with a 37°C, 5% CO_2_ chamber (Cell Imaging Centre, Faculty of Medicine and Dentistry Core Imaging Facility). Cell movement was analysed using the ImageJ software (Manual Tracking and Chemotaxis Tool plug-ins for ImageJ).

### Statistical Analysis

Statistical analysis of data was performed with a one-tailed, Student *t* test using Microsoft Excel 2011, unless otherwise indicated.

## Results

### CD45 KO BMDM Show Altered Morphology and Decreased Movement in Culture

**Figure 1:**
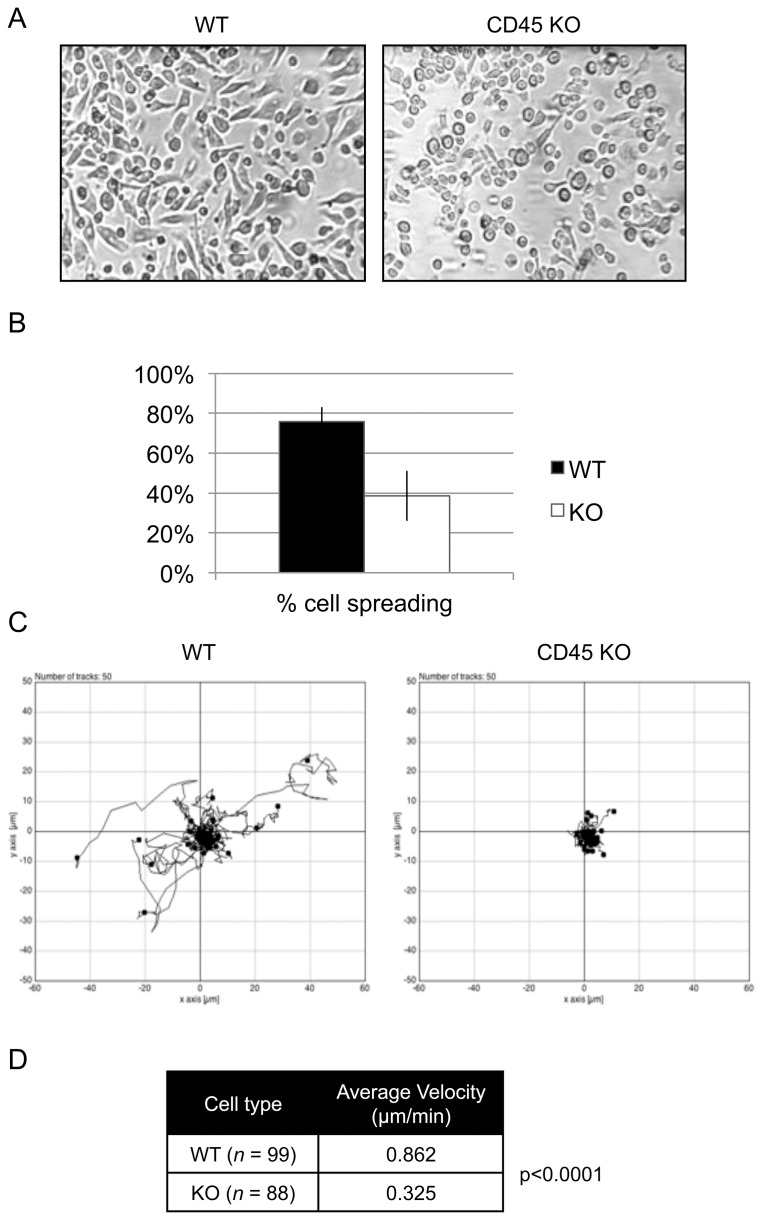
CD45 KO BMDM exhibit decreased cell spreading and motility compared to WT BMDM. (A) Morphology of day 7 WT and CD45 KO BMDM in culture. (B) Quantification of spread cells versus non-spread cells from three independent cultures of WT (black) and CD45 KO (white) BMDM as described in the materials and methods. (C) Cell tracking of fifty cells each of WT and CD45 KO BMDM on tissue-culture-treated wells for 30 minutes. (D) The average velocity of WT and CD45 KO BMDM. This data is representative of three independent experiments.

It is well known that both cell spreading and cell adhesion rely on the stable formation of focal contacts. Cell locomotion, on the other hand, relies on mechanisms that coordinate the assembly and disassembly of focal complexes. We have thus examined if these processes were affected by the absence of CD45 in macrophages. For this purpose, cell cultures were examined for cell spreading by light microscopy at day 7 of culture (Figure 1A). We found substantial differences in the morphology of macrophages derived from CD45 KO mice compared to WT mice (Figure 1B). Although CD45 KO BMDM were able to adhere to plastic surfaces during differentiation and cell culture, they showed reduced spreading and stretching when compared to WT BMDM. While the majority of WT cells showed spreading, less than half the cells in the CD45 KO BMDM culture displayed a spread phenotype. The altered morphology of CD45 KO might therefore be indicative of defects in the stability of adhesion complexes.

Live-cell imaging was then used to study cell motility of WT or CD45 KO BMDM in culture. For this purpose, BMDM were harvested at day 7 of culture and replated on TC-treated cell chambers for one hour prior to imaging. Cell movement was tracked for a period of 30 minutes and analyzed with the Chemotaxis Tool plugin of the ImageJ software (Figure 1C). WT cells stayed relatively close to their point of origin, however they still displayed detectable movement over the 30-minute recording period. Although CD45 KO cells were able to form the extensions necessary for crawling, they were unable to move and remained relatively immobile throughout the time-lapse video analysis. This is confirmed upon quantification of cell velocity of WT and CD45 KO cells, where CD45 KO macrophages display significantly less movement in culture compared to WT macrophages (p<0.0001; Figure 1D). Such reduced motility of CD45 KO macrophages may be due to several defects. First, this could be indicative of a high rate of focal complex turnover, where the disassembly of the complex occurs too rapidly for the establishment of a leading edge necessary for crawling. The decrease in cell motility could also be due to higher stability of focal structures already in place, preventing assembly of new focal structures at the leading edge of the cell. Alternatively, the high stability of established focal complexes could prevent the cells from detaching from the trailing edge of the cell. In this latter scenario, however, the cells should have displayed a stretched out morphology instead of the rounded morphology observed in Figure 1A.

Altogether, the adherence, morphology and cell motility defects observed in CD45 KO BMDM strongly suggest that disregulation of focal contact and/or focal complex stability occurs in the absence of CD45. It is thus possible that CD45 participates in the regulation of adhesion complexes through the regulation of cytoskeletal or cytoskeletal-associated proteins.

### CD45 KO BMDM Show Altered Basal Tyrosine Phosphorylation Levels

**Figure 2:**
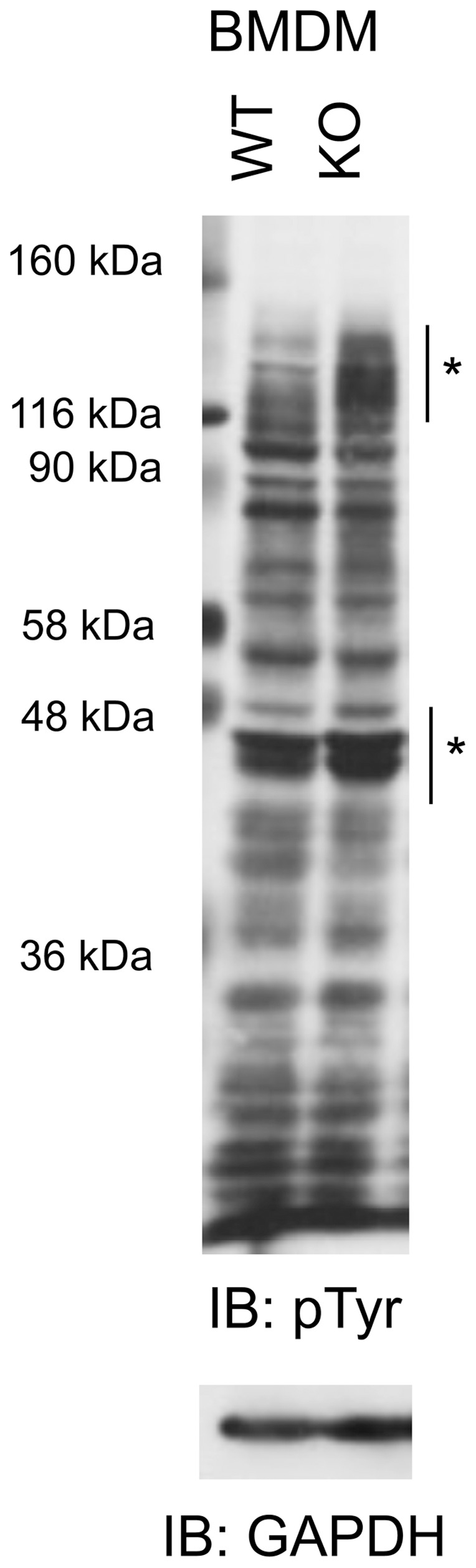
CD45 KO macrophages have limited hyperphosphorylation of proteins in lysates compared to WT. Lysates from day 7 cultures of BMDM were probed with anti-phosphotyrosine and anti-GAPDH as a loading control. Regions of the gel containing higher tyrosine phosphorylation in the CD45 KO lysates are indicated with an asterisk. Western blot is representative of more than three (BMDM) experiments. It is important to note that the molecular weight standards used migrate more slowly than predicted.

Adhesion complex assembly and disassembly in leukocytes is regulated by SFK, which are known substrates of CD45 [48], [49], [50]. CD45-deficient macrophages and T-cells have been shown to exhibit defects in adhesion, and this is thought to be SFK-dependent [5], [7], [8], [9]. Since key proteins involved in cell adhesion are subjected to regulation by SFK and the CD45 PTP, we hypothesized that these proteins would display disregulated tyrosine phosphorylation in CD45 KO BMDM. To test this, we first compared the overall tyrosine phosphorylation patterns of cellular lysate of CD45 KO and WT BMDM (Figure 2). Although the tyrosine phosphorylation of most proteins are unaffected by the loss of CD45, the tyrosine phosphorylation of a specific subset of proteins is altered in CD45 KO cells when compared to WT. In the 116–140 kDa molecular weight range, lysates of CD45 KO macrophages displayed hyperphosphorylation of multiple proteins when compared to WT lysates. Proteins in the 50 to 60 kDa range also displayed enhanced phosphorylation in CD45 KO cells. SFK, which migrate in this M.W. range, have been shown to display increased phosphorylation in CD45 KO macrophages [5], [51], which we have confirmed (data not shown). These differentially phosphorylated proteins may include proteins involved in cell adhesion and motility.

### CD45 KO BMDM Show Decreased Expression of Paxillin

**Figure 3:**
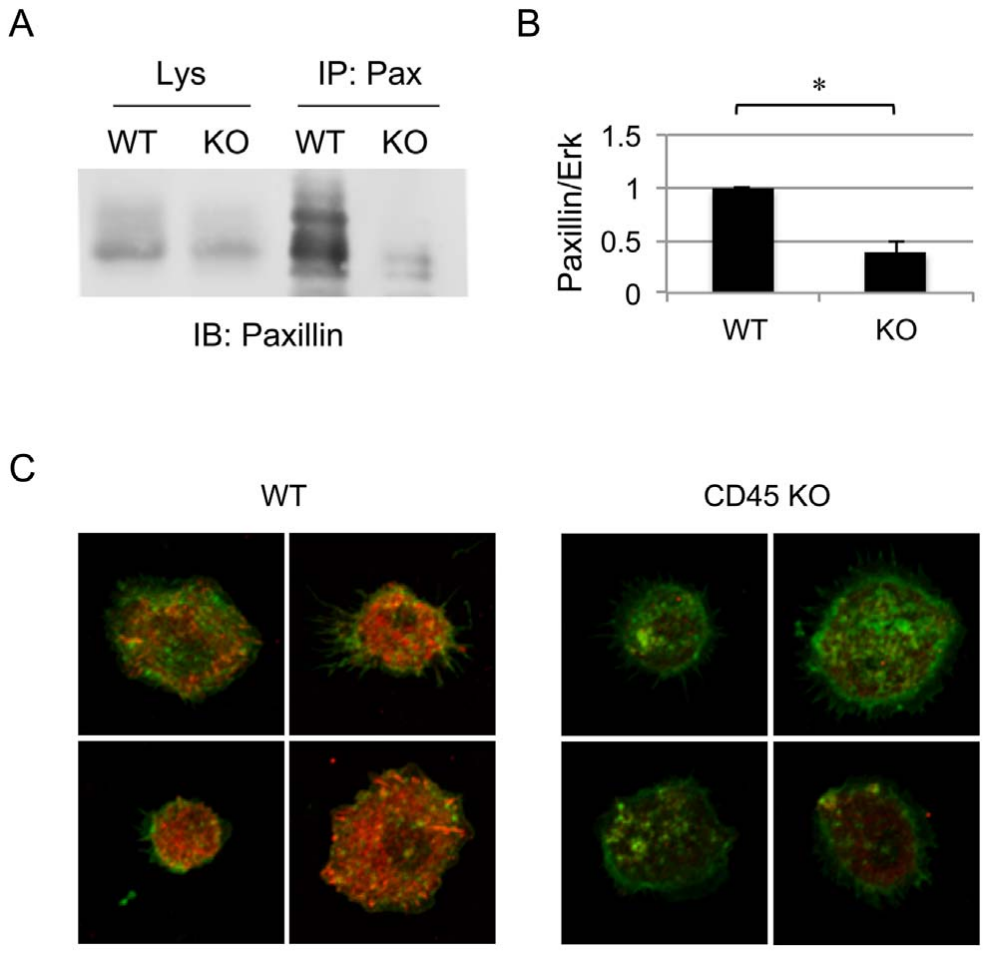
Paxillin expression is decreased in CD45 KO BMDM. (A) Paxillin was immunoprecipitated from lysates of 10^7^ day 7 WT or CD45 KO BMDM cells, followed by SDS-PAGE and Western blot with anti-paxillin. Paxillin levels were decreased in both immunoprecipitates and total lysates of CD45 KO BMDM compared to WT. Lysate control represents 4×10^5^ cell equivalents. (B) Quantification of paxillin in Western blots of BMDM lysates as represented by a ratio of paxillin in relation to the loading control (Erk). Represented is the average ratio obtained from five independent experiments. The asterisk indicates a difference with p<0.0001. (C) Immunofluorescence staining of paxillin (red) and actin (green) in day 7 CD45 KO and WT BMDM. Identical acquisition and analysis settings were used for all confocal images. Images are representative of two independent experiments.

We set out to identify proteins that are involved in macrophage adhesion and motility, are tyrosine phosphorylated and could potentially be downstream of CD45. Paxillin is a 68 kDa scaffold protein located at sites of adhesion and is tyrosine phosphorylated by FAK and Pyk2 in an SFK-dependent manner [28], [32], [33], [52], [53]. It has also been shown to be tyrosine phosphorylated upon treatment with cross-linking with an anti-CD45 Ab in T-cells [54]. We hypothesized that in CD45 KO macrophages, which harbour hyperactivated SFK, paxillin would exhibit increased tyrosine phosphorylation. To assess the effects of CD45 on paxillin phosphorylation, cellular lysates from day 7 WT or CD45 KO BMDM were immunoprecipitated and immunoblotted with anti-paxillin (Figure 3A). Such analysis, however, was complicated by the fact that CD45 KO BMDM exhibited a significant decrease in paxillin protein levels relative to a loading control (Figure 3A). This reduced expression level of paxillin was consistently observed and statistically significant (p<0.0001, using a paired t-test) compared to WT BMDM, as measured by quantitative Western blot analyses of results obtained from five independent experiments (Figure 3B).

Such decreased paxillin expression in CD45 KO BMDM was also observed by confocal microscopy (Figure 3C). In CD45 KO BMDM, paxillin staining was substantially weaker compared to that observed in WT BMDM (Figure 3C). Actin expression and localization, on the other hand, was similar between WT and CD45 KO BMDM (Figure 3C).

### Decreased Expression of Paxillin in CD45 KO BMDM is not due to Cleavage by Caspases

**Figure 4:**
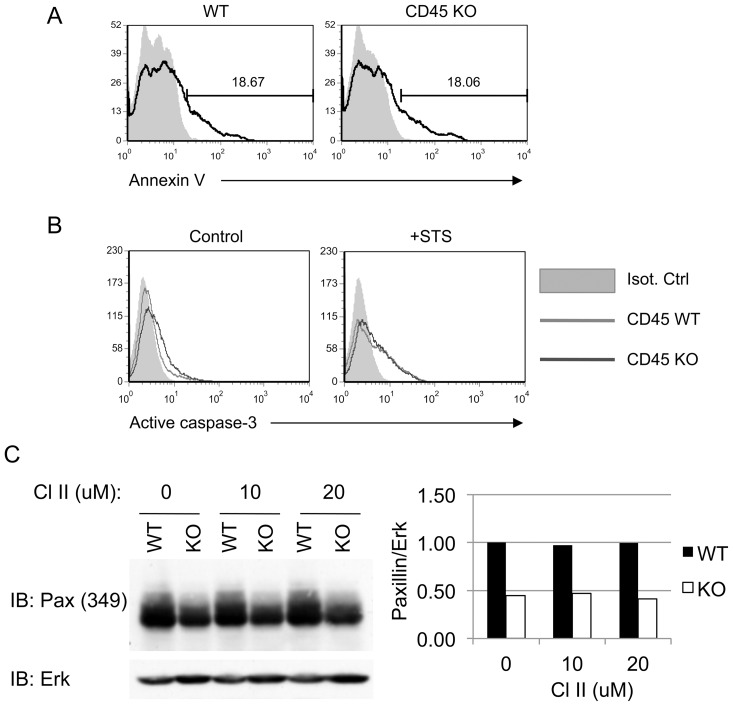
Inhibition of caspases does not restore paxillin expression in CD45 KO BMDM. (A) Unstimulated day 7 WT or CD45 KO BMDM were stained with Annexin V (black line) or left unstained (grey, filled). Both cell types exhibited similar levels of basal Annexin V staining. (B) Day 7 WT or CD45 KO BMDM were treated with 5 µM of staurosporine (STS) for 4 hours (right panel), or DMSO control (left panel), and stained with anti-active caspase-3. (C) Day 7 BMDM were treated with the indicated concentration of caspase inhibitor II (CI-II) for 4 hours and lysed. Paxillin expression was assessed by Western blot. Anti-Erk was used as a loading control. Quantification of the density of the Western blot bands was done with Image J software. The image shown is representative of three independent experiments.

Proteolytic cleavage by caspases has been shown to regulate of the turnover of several cytoskeletal-associated proteins, including paxillin, FAK and actin [55], [56], [57], [58]. This regulatory mechanism is essential for rapid dismantlement of adhesion complexes needed for adhesion turnover and cell migration. In apoptotic cells, cleavage of paxillin by caspases leads to cell rounding and detachment [57], [58], [59]. A number of studies have also showed that CD45 is known to regulate apoptosis [60], [61], [62], [63], [64], [65]. As CD45 KO BMDM display a similar phenotype to apoptotic cells in regards to cell rounding and detachment, we hypothesized that the defects in adhesion in CD45 KO BMDM resulted increased apoptosis and caspase activation, leading to paxillin degradation. To test this, day 7 BMDM from CD45 KO and WT mice were assessed for Annexin V staining and activation of caspase-3 by flow cytometry and immunoblot, respectively (Figure 4A, B). No significant differences were observed in Annexin V or anti-active caspase-3 staining between resting CD45 KO and WT BMDM. Thus, CD45 KO cells did not appear to be undergoing higher basal levels of apoptosis.

Although apoptosis did not correlate with paxillin degradation in CD45 KO BMDM, it was still possible that low levels of caspase activity resulted in paxillin degradation in CD45 KO cells without triggering obvious signs of apoptosis. For example, caspases have recently been shown to play a role in cell migration [66]. We tested this possibility by treating macrophages with CI-II, a pan caspase inhibitor, which did not restore expression of paxillin in CD45 KO macrophages (Figure 4C). It is still possible, however, that inability of CD45 KO BMDM cells to maintain adhesion may eventually lead to apoptosis, although this does not occur prior to paxillin degradation.

### Treatment of CD45 KO BMDM with a Calpain Inhibitor Restores Paxillin Expression

**Figure 5:**
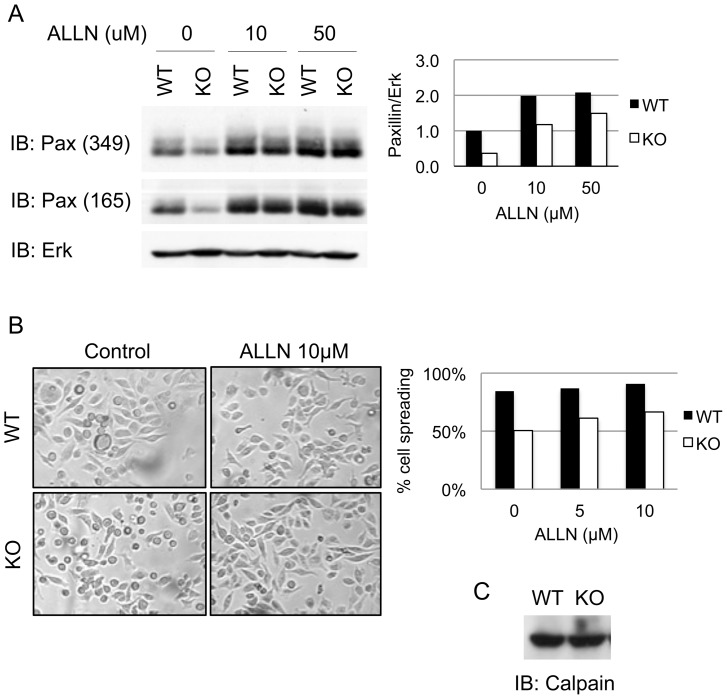
Inhibition of calpain restores paxillin expression and enhances cell spreading. (A) Day 7 WT or CD45 KO BMDM cell lysates (10^6^ cells) were treated with the indicated amounts of the calpain inhibitor ALLN for 4 hours. Lysates of 10^6^ cells were run on SDS-PAGE gel and immunoblotted with two anti-paxillin monoclonal antibodies (clones 349 and 165) and anti-Erk as a loading control. The right panel shows the quantification of paxillin expression, probed with antibody from clone 349, relative to Erk was performed with ImageJ software. (B) Light microscopy image and quantification of cell spreading of WT and CD45 KO BMDM 4 hours after treatment with 10 µM of ALLN or DMSO (control) (C). All experiments shown are representative of three independent experiments.

Calpains are calcium-dependent, non-lysosomal cysteine proteases that cleave a variety of proteins at sites of focal complexes, thereby favouring a rapid turnover of focal complexes (reviewed in [67]). Interestingly, paxillin has been shown to be sensitive to proteolysis by calpain [68], [69]. To assess whether paxillin was susceptible to proteolysis by calpain in BMDM, we treated cells with a calpain inhibitor, ALLN. We showed that a four-hour treatment of BMDM with ALLN restored paxillin expression in CD45 KO BMDM in a dose-dependent manner (Figure 5A). Paxillin expression in WT BMDM also increased with ALLN treatment, indicating that calpain-mediated paxillin turnover occurs in normal BMDM in culture. We next examined whether the restoration of paxillin expression by ALLN treatment induced changes in macrophage morphology. CD45 KO and WT cells in culture were treated for four hours with ALLN and examined by light microscopy (Figure 5B). Treatment of macrophages with ALLN increased, but did not fully restore, cell spreading in CD45 KO in a dose-dependent manner. Cell spreading was slightly increased in WT cells as well. Therefore, the increase of paxillin protein levels in CD45 KO correlated with the increase in cell spreading and stretching.

It has been shown that transformation of fibroblasts by v-Src leads to increased translation of calpain [70]. Because SFK activity is increased in the absence of CD45 [2], [4], [5], [48], we assessed whether this translated to changes in expression of calpain by Western Blot analysis. No differences in calpain expression, however, were detected between CD45 KO and WT BMDM (Figure 5C), suggesting that in CD45 KO cells, degradation of paxillin is not due to increased expression of calpain.

### Paxillin Expression and Cell Spreading is Restored in CD45 KO Macrophages upon Inhibition of Pyk2/FAK

In normal adherent cells, calpain induces the limited proteolysis of cytoskeletal-associated proteins without leading to their complete degradation [70], [71], [72]. For example, in DCs, the inhibition of calpains by ALLN prevents cleavage of Pyk2, talin and WASP without affecting their overall protein levels [73]. In the case of CD45 KO BMDM, however, calpain cleavage of paxillin leads to a decrease in its protein levels, indicating that there is amplification of this regulation mechanism to the point where there is depletion of paxillin. As calpain expression is not affected by the absence of CD45 (Figure 5C), it is possible that cleavage of paxillin by calpains is related to the disregulation of paxillin itself rather than the disregulation of calpain activity. Post-translational modifications of paxillin could lead to its increased susceptibility to cleavage. We hypothesized that tyrosine phosphorylation of paxillin may possibly contribute to its increased susceptibility to calpain cleavage. The main kinases responsible for tyrosine phosphorylation of paxillin are Pyk2 and FAK [74], [75]. These kinases are directly phosphorylated and activated by SFK, making them potential downstream targets of CD45 [22], [76]. Moreover, upon CD44-mediated cell spreading of T-cells, Pyk2 and FAK exhibit increased phosphorylation in CD45 KO cells [8], [9]. This increased Pyk2 and FAK phosphorylation is SFK-dependent.

**Figure 6:**
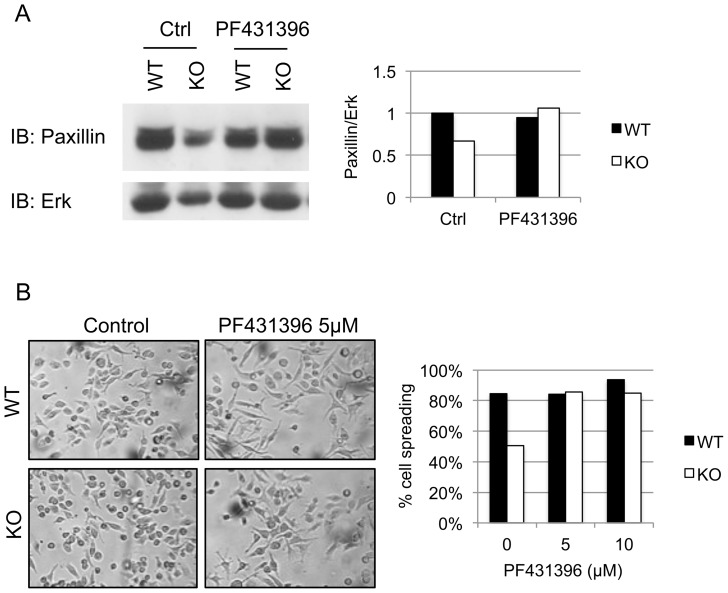
Inhibition of Pyk2 and FAK restores paxillin expression and cell spreading. (A) Day 7 WT or CD45 KO BMDM were treated with 5 µM of the Pyk2/FAK inhibitor PF431496, or DMSO carrier, for 4 hours. Lysates were run on SDS-PAGE gel and immunoblotted with anti-paxillin and anti-Erk as a loading control. Also shown is the quantification of the band density of this experiment. (B) Light microscopy and cell spreading quantification upon treatment with PF431396, or DMSO as control, for 4 hours. Data is representative of three independent experiments.

To examine the contribution of Pyk2 and FAK in paxillin proteolysis, cells were treated for 4 hours with the Pyk2/FAK inhibitor PF431396. This inhibitor prevents ATP binding and kinase activity of both Pyk2 and FAK [77]. Upon inhibition of Pyk2 and FAK, paxillin expression was restored in CD45 KO BMDM (Figure 6A). Indeed, prior to treatment, paxillin expression was significantly different between WT and CD45 KO BMDM (p<0.01) whereas differences in paxillin expression between WT and CD45 BMDM after treatment with 5 µM of PF431396 were not. This indicates that tyrosine phosphorylation of paxillin by one, or both, of these kinases promotes its cleavage by calpains.

This experiment also provided an opportunity to test whether restoration in paxillin expression resulted in changes in macrophage morphology. For this purpose, we examined cell morphology of CD45 KO or WT BMDM by light microscopy after a 4-hour treatment with increasing concentrations of PF431396 (Figure 6B). We found that even at low concentration, treatment with PF431396 restored a morphological phenotype in CD45 KO BMDM that was similar to that of WT macrophages. Such morphological changes correlated with the restoration of paxillin expression. The changes in morphology were more pronounced than what was observed with ALLN (Figure 5B). This could be due to non-paxillin-dependent adhesion events that are negatively regulated downstream of Pyk2/FAK. Nonetheless, we provide evidence that the CD45-dependent regulation of paxillin degradation by calpains is Pyk2/FAK-dependent.

## Discussion

Herein we uncovered a potential molecular mechanism that explains the contribution of CD45 to the regulation of cell adhesion, morphology and motility in macrophages. More specifically, we found that 1) CD45 KO BMDM showed defects in cell spreading and motility, 2) the absence of CD45 led to an increase in calpain-mediated cleavage of paxillin, and 3) the degradation of paxillin was substantially reduced with an inhibitor of Pyk2/FAK. Together, these results provide evidence that in macrophages, tyrosine phosphorylation events downstream of CD45 play a central role in their adhesion and migration functions.

The inability of macrophages to maintain adhesion to ECM components in the absence of CD45 has been previously demonstrated using CSF-derived BMDM [78]. Here we show a similar effect of CD45 on adhesion using GM-CSF-derived BMDM suggesting that CD45 regulates adhesion in both types of macrophages. We also showed that CD45 also regulates cell morphology and cell motility. Although the small round cellular phenotype that we observed in the CD45-deficient macrophages is typical of cells undergoing apoptosis, no signs of apoptosis (such as elevated levels of AnnexinV staining and activation of caspase-3) were detected in cultured CD45 KO BMDM. These results are thus consistent with a model whereby CD45 regulates specific signalling pathways that normally control the turnover of adhesion structures, whether during assembly or their disassembly, or both.

In order to uncover the mechanisms underlying CD45-dependent adhesion regulation, we first assessed differences in tyrosine phosphorylation (Figure 2). In addition to the hyperphosphorylation of SFK in CD45 KO cells, which has been established in the literature [5], [51], we have observed hyperphosphorylation of several proteins in the 116–140 kDa MW range. Although we have not determined the identity of these proteins, their identification and the role they play in CD45-regulated cell adhesion is an interesting avenue for future investigations, particularly because several SFK-regulated cytoskeletal-associated proteins migrate in this MW range, including FAK (125 kDa), Pyk2 (112–116 kDa), p130Cas (130 kDa), and vinculin (145 kDa). Therefore, it is thus logical to predict that some of these proteins are potential downstream targets of CD45 during macrophage adhesion.

In the present work, we focussed on the cytoskeletal-associated protein paxillin as a potential downstream target of CD45. The rationale was based on the knowledge that paxillin is phosphorylated by SFK and plays a key role in the assembly and turnover of adhesion structures. We initially hypothesized that paxillin would be hyperphosphorylated in CD45 KO BMDM. This hypothesis could not be tested, however, because paxillin expression was drastically reduced in CD45 KO BMDM. These results led to further investigation on the role of caspases and calpains, both of which have been shown to regulate adhesion complex turnover by cleaving cytoskeletal-associated proteins including paxillin. Using specific pharmacological inhibitors of calpain and caspases, we found that calpains, rather than caspases, were responsible for paxillin degradation in CD45 KO BMDM. Restoration of paxillin expression also increased cell spreading in CD45 KO BMDM. Inhibition of calpains in WT cells also increased paxillin expression and cell adhesion. We thus propose a model in which during cellular adhesion and deadhesion in WT macrophages, paxillin protein levels are not overtly affected by calpain cleavage. This is possibly due to the fact that the fraction of paxillin cleaved by calpains is replaced by *de novo* synthesis. In the absence of CD45, however, cleavage of paxillin is enhanced and *de novo* synthesis may not occur rapidly enough to compensate for this loss in paxillin proteins levels, leading to its depletion in CD45 KO BMDM.

We then sought to identify by which means CD45 could promote paxillin degradation. We hypothesized that CD45 could regulate paxillin degradation either by the activation of cellular calpains, or through the regulation of paxillin itself. In the former scenario, for example, CD45 could regulate calpain activation through the control of intracellular calcium mobilization or through direct activation of calpains by Erk phosphorylation. However, we do not observe the degradation of other proteins known to be targets of calpain, such as Pyk2 itself [73], [79]. In the latter scenario, phosphorylation of paxillin could lead to conformational changes in the protein, leading to exposed sites of calpain cleavage. This could occur either through serine/threonine phosphorylation which is mediated by MAP kinases, or through tyrosine phosphorylation mediated by Pyk2, FAK and SFK. Both the MAP kinases and the tyrosine kinases Pyk2, FAK and SFK have been shown to be regulated by CD45 and may be disregulated in the absence of this protein [8], [9], [41], [80], [81], [82], [83], [84].

We assessed whether tyrosine phosphorylation of paxillin would increase its susceptibility to calpain cleavage. FAK is involved in the regulation of paxillin phosphorylation, and this step is critical for focal adhesion assembly [85]. This may occur due to direct phosphorylation of paxillin by FAK, or by bringing paxillin and Src in close proximity, leading to phosphorylation of paxillin by Src [53], [85]. Pyk2, a relative of FAK, can also associate with paxillin and is thought to regulate paxillin phosphorylation in a similar manner [33], [86], [87], [88]. We hypothesized that these kinases were involved in the regulation of paxillin degradation through tyrosine phosphorylation of paxillin. Upon treatment of BMDM with a Pyk2/FAK inhibitor, PF431396, paxillin expression and cell spreading were restored in CD45 KO BMDM. Since this inhibitor blocks the kinase activity of both Pyk2 and FAK, the ability of these kinases to phosphorylate substrate is therefore required for paxillin degradation [77]. It is thus possible that tyrosine phosphorylation of paxillin by Pyk2 and/or FAK increases the susceptibility of paxillin to calpain-mediated cleavage. Interestingly, the major calpain cleavage site on paxillin (S95) is found in between the two major sites of tyrosine phosphorylation in paxillin (Y31 and Y118). It is conceivable that phosphorylation of paxillin at these sites promotes a conformation of the protein in which the calpain cleavage site is exposed. As calpains and phosphorylated paxillin are both found at sites of adhesion, proteolysis of paxillin at these sites may lead to focal complex disassembly and ultimately, to cell rounding and detachment. This model provides an additional mechanism by which Pyk2 and/or FAK regulate adhesion turnover.

Inhibition of paxillin calpain-mediated degradation by the Pyk2/FAK inhibitor may also be indirect, as treatment with this inhibitor may stabilize focal contacts and prevent paxillin turnover. This is supported by the fact that treatment of macrophages and T-cells with inhibitor renders cells increasingly adherent (Figure 6B and Cheung and Ostergaard, unpublished observation).

Our data suggests that increased SFK activity in CD45 KO cells would lead to increased phosphorylation and activity of Pyk2 and/or FAK, both of which are direct substrates of SFK and are capable of phosphorylating paxillin. Increased activity of Pyk2 and/or FAK could in turn lead to an increase in paxillin phosphorylation and subsequent proteolysis. We showed here that Pyk2/FAK inhibition restores paxillin expression in CD45 KO BMDM (Figure 4.12a), supporting the link between CD45, Pyk2/FAK and paxillin. Finally, we showed that Pyk2/FAK inhibition promotes cell spreading in CD45 KO BMDM (Figure 4.12b). Further experiments will be needed in order to elucidate the contribution of Pyk2 and/or FAK to this process.

In summary, the results herein have revealed a yet undescribed mechanism by which CD45 regulates macrophage adhesion, morphology and motility. This mechanism is based on the ability of CD45 to regulate calpain-mediated cleavage of paxillin in a Pyk2/FAK-dependent manner. Further understanding of this pathway will be important in the identification of methods to regulate macrophage adhesion and migration in disease.
